# Effect of repeated hot water immersion on cognitive performance, cerebrovascular function, sleep and biomarkers of neurodegeneration in older adults

**DOI:** 10.1113/EP093500

**Published:** 2026-04-29

**Authors:** Daniel D. Piccolo, Jo Corbett, Joseph T. Costello, Thomas B. Williams, Thomas J. James, Janis K. Shute, Mohammad G. A. Alnajjar, Luke C. Hudson, Poppy A. Marsh, Veronika Praskacova, Harry S. Mayes, Michael Tipton, Maria Perissiou, Melitta A. McNarry, Kelly A. Mackintosh, Zoe L. Saynor, Anthony I. Shepherd

**Affiliations:** ^1^ Clinical, Health and Rehabilitation Team, Centre for Integrated Health and Wellbeing, School of Psychology, Sport, and Health Sciences, Faculty of Science and Health University of Portsmouth Portsmouth UK; ^2^ Extreme Environments Laboratory, School of Psychology, Sport, and Health Sciences, Faculty of Science and Health University of Portsmouth Portsmouth UK; ^3^ Cardiovascular Health Sciences Research Group Liverpool John Moores University Liverpool UK; ^4^ School of Medicine, Pharmacy and Biomedical Science, Faculty of Science and Health University of Portsmouth Portsmouth UK; ^5^ Institute of Marine Sciences Faculty of Science and Health University of Portsmouth Portsmouth UK; ^6^ Applied Sports, Technology, Exercise and Medicine (A‐STEM) Research Centre, School of Sport and Exercise Sciences Swansea University Swansea UK; ^7^ School of Health Sciences Faculty of Environmental and Life Sciences University of Southampton Southampton UK; ^8^ National Institute for Health Research Southampton Biomedical Research Centre Southampton UK

**Keywords:** ageing, exercise mimetic, passive heat therapy, working memory

## Abstract

Ageing is associated with cognitive decline and increased risk of developing neurodegenerative disease. Repeated passive heating, using hot water immersion (HWI), may improve cognitive performance via improved cerebral oxygenation, but this is yet to be examined in older adults. Twelve healthy older adults (aged: 69.2 ± 10.0 years; body mass index: 25.2 ± 4.1 kg m^−2^) completed a 6‐week pre–post intervention study consisting of two to three weekly 1 h HWIs in 40°C water. Rectal temperature was maintained in a target range of 38.5–39.0°C during HWI. Cognitive performance (working memory via 1 and 2‐back, inhibition via 2‐choice reaction time, logical reasoning via logical relations) and cerebral oxygenation (Δoxyhaemoglobin, Δdeoxyhaemoglobin, Δtotal haemoglobin and Δtissue saturation index) were assessed during the first and final HWI sessions (pre‐, immediately post‐ and 3 h post‐HWI). Common carotid artery blood flow (CCA‐BF), sleep quality (7‐day baseline and final week), plasma [amyloid‐β] 42 (Aβ42), and [phosphorylated tau] (p‐tau), were measured pre‐ and post‐intervention. Repeated HWI improved 1‐back (*P* = 0.023) and logical reasoning (*P* = 0.002) performance, but not 2‐back or 2‐choice reaction time (*P* > 0.05). Cerebral oxygenation was acutely reduced immediately post‐HWI (all parameters *P* < 0.05), but returned to baseline 3 h post‐HWI, with no chronic adaptation. CCA‐BF, sleep quality, [Aβ42] and [p‐tau] all remained unchanged at 6 weeks (*P* > 0.05). Repeated HWI improves cognitive domains of logical reasoning and working memory without altering cerebral oxygenation, CCA‐BF, sleep or neurodegenerative biomarkers. Further investigation into the underlying mechanisms for cognitive performance improvements via HWI is warranted.

ageing, exercise mimetic, passive heat therapy, working memory

## Abstract (highlights)

- **What is the central question of this study?**
  Can 6 weeks of hot water immersion improve cognitive performance, cerebrovascular function, sleep and neurodegenerative biomarkers in healthy older adults?
- **What is the main finding and its importance?**
  Six weeks of two to three hot water immersions per week improved working memory and logical reasoning in healthy older adults, but did not alter common carotid artery blood flow or oxygenation, sleep, or neurodegenerative biomarkers. The results suggest that hot water immersion may offer a simple, non‐pharmacological, therapeutic approach to support cognitive performance in older adults, though mechanisms remain to be clarified.

## 1 INTRODUCTION

Ageing is accompanied by progressive declines in cognitive function (Harada et al., 2013), which manifest as impaired memory (Harada et al., 2013), reaction time (Fozard et al., 1994) and reasoning (Singh‐Manoux et al., 2012). These deficits are primary risk factors for developing neurodegenerative disease (Hou et al., 2019), which in turn reduces daily function (i.e., performing activities of daily living) (Andersen et al., 2004). Ageing also impairs neurovascular coupling (Tarantini et al., 2017), reduces resting cerebral blood flow (CBF) (Stoquart‐ElSankari et al., 2007) and reduces sleep quality (Ohayon et al., 2004), all of which are linked to impaired cognition (Miyata et al., 2013; Rabbitt et al., 2006; Tarantini et al., 2017). With the global population of older adults aged ≥65 years expected to reach 2.2 billion by 2080 (United Nations, 2024), effective interventions to preserve or enhance cognitive health are urgently needed.

Passive heat therapy (PHT), such as sauna bathing or hot water immersion (HWI), has emerged as a potential non‑pharmacological strategy to support brain health (Hunt et al., 2020; von Schulze et al., 2020). While acute elevations in core temperature above 38.5°C can impair complex cognitive performance (Schmit et al., 2017), previous repeated PHT studies in young, healthy adults have been shown to prevent declines in, or improve, working memory (Racinais et al., 2017) and rapid visual processing (Radakovic et al., 2007) immediately following acute heat stress. However, repeated PHT does not appear to alter baseline cognitive function in young, healthy adults (Barry et al., 2022; Racinais et al., 2017; Radakovic et al., 2007), likely due to ceiling effects. In contrast, older adults typically exhibit reduced cognitive function (Harada et al., 2013), and may have more capacity to improve. Supporting this, longitudinal population‐based studies report strong associations between frequent sauna use and reduced risk of dementia (Knekt et al., 2020; Laukkanen et al., 2017) and Alzheimer's disease (Laukkanen et al., 2017) in older adults.

One proposed mechanism by which PHT could enhance cognitive function is increased CBF. This could subsequently improve cerebral oxygenation, which is linked to cognitive performance (Williams et al., 2019) and is often reduced in older adults during brain activation (Schroeter et al., 2003). However, the reported effects of PHT on CBF are inconsistent. PHT using water‐perfused suits (Crandall & Wilson, 2015) and lower‐body HWI (Ota et al., 2019) have demonstrated acute decreases in CBF, whereas whole‐body HWI appears to acutely increase CBF (Gibbons et al., 2021; Worley et al., 2021), likely via maintained end‐tidal carbon dioxide ($P_{\mathrm{ETC}O_{2}}$) levels during immersion (Gibbons et al., 2021). Repeated exposure may enhance vascular endothelial function (Bailey et al., 2016), providing another contributory mechanism. To date, however, no study has investigated the effects of repeated whole‐body HWI on CBF in older adults.

At the molecular level, age‐related accumulation of amyloid‐β (Aβ) (Thal et al., 2006) and phosphorylated tau (p‐tau) (Delacourte et al., 1999) deposits in the brain contribute to cognitive decline and the development of neurodegenerative diseases (Sperling et al., 2019). HWI can induce increased concentrations of cytoprotective heat shock proteins (Brunt et al., 2018), which may mitigate against harmful protein aggregation (Lindquist & Craig, 1988), offering another potential mechanism for neuroprotection. Whilst HWI may upregulate molecular protections that could aid cognitive performance, HWI could also benefit older adults’ sleep quality. Acute HWI in the afternoon and evening has improved sleep quality in younger (Horne & Reid, 1985) and older adults (Bunnell et al., 1988; Liao, 2002), while sauna users also report higher sleep satisfaction (Engström et al., 2024) and acute improvements in sleep quality (Hussain et al., 2019). However, no repeated laboratory studies exploring the effects of PHT on sleep and subsequent links with cognitive performance have been undertaken.

This study therefore aimed to examine the acute and repeated effects of HWI on cognitive performance, cerebral oxygenation, common carotid artery blood flow (CCA‐BF), sleep and circulating biomarkers of brain health in healthy older adults. We hypothesised that acute HWI would transiently reduce complex cognitive performance and cerebral oxygenation immediately following HWI, but that repeated (6 weeks) HWI would attenuate these reductions. Moreover, we hypothesised that repeated HWI would (i) improve cognitive performance pre‐HWI, (ii) increase resting CCA‐BF, (iii) improve sleep quality and efficiency, and (iv) reduce [Aβ] and [p‐tau].

## 2 METHODS

### 2.1 Ethical approval

This open label pre–post experimental trial received approval from the University of Portsmouth Faculty of Science and Health Ethics Committee (SHFEC: 2022–054). The study was conducted in accordance with the *Declaration of Helsinki*, and was pre‐registered on ClinicalTrials.gov (ID no. NCT05618197). Participants received a participant information sheet ≥48 h prior to enrolment. They were then fully briefed on the study procedures and given the opportunity to ask any questions before providing fully informed written consent.

### 2.2 Participants

Participants were recruited through media releases, local interest groups, local databases, posters and word of mouth. Inclusion criteria were age ≥55 years, and absence of any cardiometabolic disease or neurological condition that would cause them to be unable to complete the cognitive tasks. Potential participants were excluded if they had a body mass index >35 kg m^−2^, uncontrolled hypertension (≥150 mmHg systolic and/or ≥90 mmHg diastolic). Further exclusion criteria were if participants were a current or recent smoker (in the last three months) and/or had prolonged exposure to a hot climate in the previous 3 months.

The primary outcome was the change in working memory performance, a major symptom of neurodegenerative diseases (Hou et al., 2019), assessed immediately following HWI via an 2‐back cognitive test before the first and final HWI session. An a priori sample size calculation indicated that seven participants were needed to detect a change in working memory performance (*n*‐back level) with 90% power and an α of 0.05, based on an effect size of *d* = 1.67 from Laine et al. (2018). The present study was part of a larger trial with smaller effect sizes for other outcome measures. The larger trial aimed to complete with 13 participants, and therefore we sought to recruit 20 people, to account for potential dropouts.

### 2.3 Study overview

**FIGURE 1:**
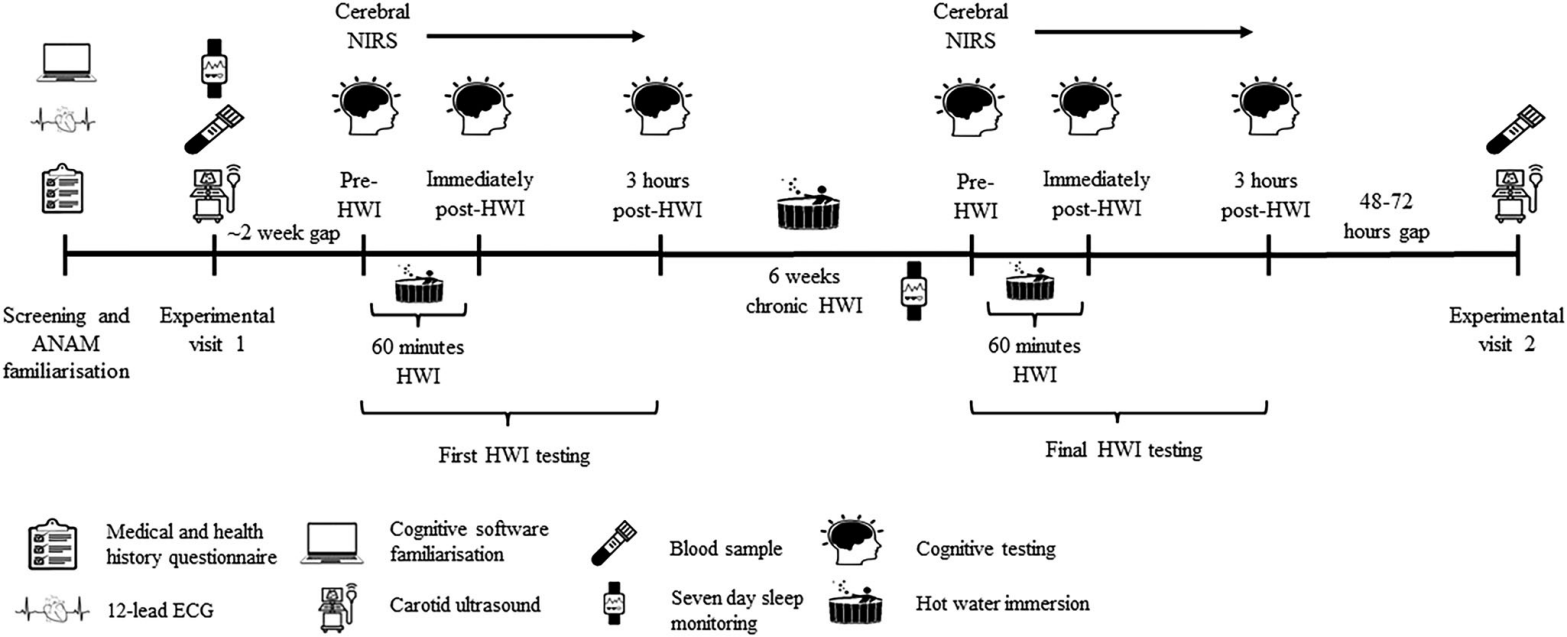
Schematic of study timeline. ANAM, automated neurophysiological assessments metrics; ECG, electrocardiogram; HWI, hot water immersion; NIRS, near‐infrared spectroscopy.

This study employed a pre–post design, which involved an initial screening visit, an experimental visit before and after a minimum 12 and maximum 18 HWIs (two to three per week) over a 6‐week period (between May 2023 and July 2025), and acute cognitive testing on two days, during both the first and final HWI sessions (Figure 1). The screening visit included completion of a health history questionnaire, height and body mass measurements, baseline blood pressure, a 12‐lead electrocardiogram, and familiarisation with the cognitive testing software. Participants were given an accelerometer to wear on their wrist for 7 days to monitor sleep on two occasions: before undertaking their first HWI and during the final week of the intervention period. Cognitive testing during the first and final HWIs was conducted across three time periods: pre‐HWI, immediately post‐HWI and 3 h post‐HWI, where previous research in our laboratories has shown physiological variables (e.g., heart rate and blood pressure) to return to baseline following HWI (James et al., 2021). Experimental visit 1 was conducted 2 weeks ± 2 days before the first HWI while the post‐intervention experimental visit took place 48–72 h after the final HWI session.

### 2.4 Protocol

#### 2.4.1 Experimental visits

Participants arrived at the laboratory in the morning (∼10.00 ± 2 h) having abstained from heavy exercise and alcohol for 24 h and caffeine on the morning of the visit. Following a 10‐min supine rest in ambient conditions (23°C, 50% relative humidity), measurement of cerebral blood flow was recorded, and a venous blood sample was taken (described below).

#### 2.4.2 Intervention (HWI)

Participants completed two to three HWI sessions per week for 6 weeks, broadly in line with previous studies (Brunt et al., 2016; Hesketh et al., 2019). Each HWI session involved immersion to just below shoulder height, in 40°C water, for 60 min in a hot tub (Hawaii, Lay‐Z‐Spa, Newton Abbot, UK) or temperature‐controlled immersion pool within the Extreme Environments Laboratory (University of Portsmouth). The first HWI session took place in the morning (10.00 ± 1 h), while the final HWI was conducted at 11.00 ± 2 h, which suited participant availability. Rectal temperature (*T*_rec_) was continuously monitored (2040 Squirrel, Grant Instruments, Cambridge, UK) for the 60 min HWI and until participants’ *T*_rec_ cooled <38.5°C afterwards using a self‐inserted rectal thermistor (Rectal temperature probe, Philips, Netherlands or YSI 400 series temperature probe, PROACT Medical, Corby, UK). Each participant used the same model of rectal thermistor throughout the trial. Body position was adjusted (i.e., participants were lifted out of or put back in the water) when necessary to ensure the target *T*_rec_ of 38.5–39.0°C was maintained throughout each immersion as described in our previous work (James et al., 2023). Heart rate was continuously monitored with a commercially available strap and watch (FT1, Polar Electro Oy, Kempele, Finland), and blood pressure was measured every 15 min (M3, Omron, Kyoto, Japan), to ensure safety during each HWI.

### 2.5 Outcome measures

#### 2.5.1 Cognitive tasks

Cognitive performance was assessed using a series of computerised tasks administered via the Automated Neurophysiological Assessments Metrics (ANAM) system (Cognitive Science Research Center, University of Oklahoma, OK, USA), and delivered using a laptop computer (Acer TravelMate P, Acer, New Taipei City, Taiwan). We assessed inhibition via a two‐choice reaction time, logical reasoning via a logical relations task and working memory via running memory continuous performance (1‐ and 2‐back), as previously described and used within our laboratories (Williams et al., 2024).

The ANAM software has previously been shown to have good construct validity (Short et al., 2007) and excellent test–retest reliability (Vincent et al., 2018). To reduce the occurrence of a learning effect, participants completed a minimum of three familiarisation trials, in line with the manufacturer's recommendations (ANAM, 2015). Before each series of tasks began, participants were instructed to respond as accurately and as quickly as possible. The key outcome measure for each cognitive task was throughput. Throughput is considered a measure of effectiveness of cognitive efficiency and is reported as the number of correct responses per minute of available response time (higher values indicate better performance) (Thorne, 2006).

#### 2.5.2 Cerebral oxygenation

Cerebral oxygenation was non‐invasively measured using near‐infrared spectroscopy (NIRS) (Portalite, Artinis Medical, Elst, The Netherlands), which detects changes in oxygen‐dependent light absorption by oxy‐ and deoxyhaemoglobin through three optodes at depths of 30 (T1), 35 (T2) and 40 mm (T3), respectively. The NIRS device was used to measure oxygenation of the prefrontal cortex at wavelengths between 760 and 850 nm. The Portalite was attached on the forehead to the surface of the left prefrontal cortex, between Fp1 and F3 (international EEG 10–20 system) (Jasper, 1958), and was worn for the entirety of the first and final HWI trials. The measurement site was cleaned with an alcohol wipe, and subsequently covered with a transparent film prior to placement of the device. To protect from light interference, the device was shielded using an opaque tape, or black cloth and headband. Measurements included an age‐dependent differential path‐length factor (DPF) according to the manufacturer's guidelines (Duncan et al., 1996). After attaching the NIRS device, a resting 2‐min baseline was recorded with the participant in a semi‐recumbent position on a bed, before the participant then undertook baseline cognitive testing. Participants continuously wore the NIRS device throughout the 60 min HWI and until the subsequent two cognitive testing batteries were completed, immediately post‐HWI and 3 h post‐HWI, respectively. All NIRS data were then expressed as changes from baseline (Δ) (Komiyama et al., 2017), including oxyhaemoglobin (ΔO_2_Hb), deoxyhaemoglobin (ΔHHb), total haemoglobin (ΔtHb) and tissue saturation index (ΔO_2_Hb/ΔtHb × 100%), which were calculated for the periods when participants were completing the cognitive testing. To limit the influence of extracranial signal contamination in the NIRS data at optode depths of 30 mm that may occur with increases in skin blood flow (Hirasawa et al., 2015), superficial signals (T1) were subtracted from the deepest signals (T3) (Saager & Berger, 2008) for measurements of ΔO_2_Hb, ΔHHb and ΔtHb. While completing the cognitive tasks, participants were instructed to keep their head as still as possible to reduce NIRS measurement error from head movement.

#### 2.5.3 CCA‐BF

To obtain CCA‐BF, the right common carotid artery was imaged at rest for 60 s using a 15.0–4.0 MHz linear wideband array ultrasound probe attached to a portable ultrasound system (Terason uSmart 3300, Burlington, MA, USA), with a 60° insonation angle following previous recommendations (Stein et al., 2008). The image depth was 4 cm, with a single focal zone. The location of the transducer for each scan was recorded via a tape measure and photograph for repeat scans within participants. All scans were performed by the same member of the research team (D.D.P.). Simultaneous recordings of CCA diameter and blood velocity were made continuously using screen recording software (Camtasia Studio, Techsmith, East Lansing, MI, USA) and analysed using custom edge detection and wall‐tracking software (Quipu Cardiovascular Suite Ver 5.0.0, Pisa, Italy). Blood velocity measures were averaged over a 60 s period as described by Gibbons et al. (2021). CCA‐BF was calculated as the product of the mean blood velocity and vessel cross‐sectional area (Volumetric flow rate ($\dot{Q}$) = blood velocity (*v*) × cross‐sectional area ($A=\pi {\left(d/2\right)}^{2}$) (Turner et al., 2024). Test–retest reliability (coefficient of variation) for this operator using this software was 1.7% for diameter, 8.1% for blood velocity and 7.7% for blood flow (*n *= 6).

#### 2.5.4 Sleep

Sleep profiles were assessed at 100 Hz using a GENEActiv (Activinsights, Kimbolton, Cambridge, UK) accelerometer, a wrist worn device that is valid and reliable for measuring sleep in adults (Van Hees et al., 2015). Specifically, assessments were made over two separate 7‐day periods during the study. Participants wore the accelerometer on the same wrist for a baseline 7‐day period before the first HWI session, and during the final week of HWI. Following the measurement periods, data were downloaded using the manufacturer's software and processed in R (version 4.2.2., R Core Team, Vienna, Austria) using an open‐source sleep detection algorithm in the GGIR software package (https://cran.r‐project.org/web/packages/GGIR). Sleep metrics derived using this method have demonstrated good levels of agreement with both self‐reported measures of sleep and gold standard polysomnography (Full et al., 2018). The method of accelerometer‐based sleep quantification used in this study is described in detail elsewhere (Van Hees et al., 2015). Time in bed was defined as the onset of the first period of sustained inactivity (as measured by changes of less than 5 degrees in a rolling 5 min window) to the end of the last period of inactivity. Sleep duration is the sum of all recorded periods of sleep. Sleep efficiency was then calculated as the sleep duration as a proportion of time in bed. Participants who recorded <four days with 16 h wear‐time were excluded.

#### 2.5.5 Blood samples

Venous blood was drawn from the antecubital fossa into EDTA tubes (K2, BD Biosciences, San Jose, CA, USA). Haemoglobin (201+, HemoCue, Ängelholm, Sweden) and haematocrit (microhaematocrit capillary tubes, Brand, Wertheim, Germany) were measured in triplicate from the EDTA sample in whole blood to assess plasma volume changes (Dill & Costill, 1974). EDTA tubes were then spun in a chilled (4°C) centrifuge (Megafuge ST Plus, Thermo Fisher Scientific, Waltham, MA, USA) at 4500 *g* for 10 min to separate plasma for [Aβ] and [p‐tau] analysis. Plasma was subsequently aliquoted into Eppendorf tubes and stored at −80°C.

#### 2.5.6 Biochemical analysis

Plasma [Aβ42] (ab289832; Abcam, Cambridge, UK), and [p‐tau] (ab273617; Abcam) were analysed in duplicate using commercially available enzyme‐linked immunosorbent assay kits and analysed using a plate reader (SpectraMax i3x; Molecular Devices, Winnersh, UK) (intraplate CV; Aβ42 = 6.5%, p‐tau = 5.7%).

### 2.6 Data analysis

Statistical analyses were conducted using SPSS Statistics, version 30.0 (IBM Corp., Armonk, NY, USA). Data normality was assessed using descriptive methods (skewness, outliers, and distribution plots) and inferential statistics (Shapiro–Wilk test). Data are presented as means ± standard deviations (SD) unless otherwise stated, with statistical significance set at *P* < 0.05. Grand means ± SD (i.e., all time points added together and averaged) were calculated for *T*_rec_, as well as all three time points (pre‐HWI, immediately post‐HWI and 3 h post‐HWI) of cognitive performance assessment and cerebral oxygenation measurements during the first HWI and final HWI.

The primary outcome, change in 2‐back performance between the first and final HWI session immediately post‐HWI, was assessed using a paired samples Student's *t*‐test. Main and interaction effects for additional cognitive measures and NIRS data were assessed using a 2 × 3 linear mixed model [Condition (first HWI and final HWI) × Time (pre‐HWI, immediately post‐HWI and 3 h post‐HWI)] to account for missing data points (1‐back in final HWI immediately post‐HWI for *n* = 1; NIRS data in first HWI immediately post‐HWI for *n* = 1; NIRS data in first HWI 3 h post‐HWI for *n* = 2; NIRS data in final HWI at 3 h post‐HWI for *n* = 2). Significant interactions were followed by *post hoc* analysis with Fisher's least significant difference test. Statistical differences for all grand means and remaining outcome variables were assessed by paired samples *t*‐test or Wilcoxon signed‐rank test (for non‐parametric data). For linear mixed model data, effect sizes were reported as partial eta squared (η^2^_p_; small = 0.01, medium = 0.06 and large = 0.14) (Lakens, 2013). Measures for the paired samples *t*‐test effect size were reported as Cohen's *d* (small = 0.2, medium = 0.5, and large = 0.8) (Cohen, 2013). Measures for the Wilcoxon signed‐rank test effect size were reported as Rosenthal's *r* (weak = 0.2, moderate = 0.4, and strong = 0.6). Pearson's and Spearman's correlations were performed for parametric and non‐parametric data, respectively, classified as negligible (*r* = 0.00–0.10), weak (*r* = 0.10–0.39), moderate (*r* = 0.40–0.69), strong (*r* = 0.70–0.89), and very strong (*r* = 0.90–1.00), respectively. ANAM throughput scores for the penultimate and final familiarisation trials for each participant were analysed using paired samples *t*‐tests or Wilcoxon signed‐rank tests. Biomarkers were adjusted for plasma volume changes using the Dill and Costill (1974) method. As the DPF has only been validated for adults aged 18–50, the value for age 50 was used for all participants, and effect sizes were used to determine any changes in NIRS (Schroeter et al., 2003).

## 3 RESULTS

**FIGURE 2:**
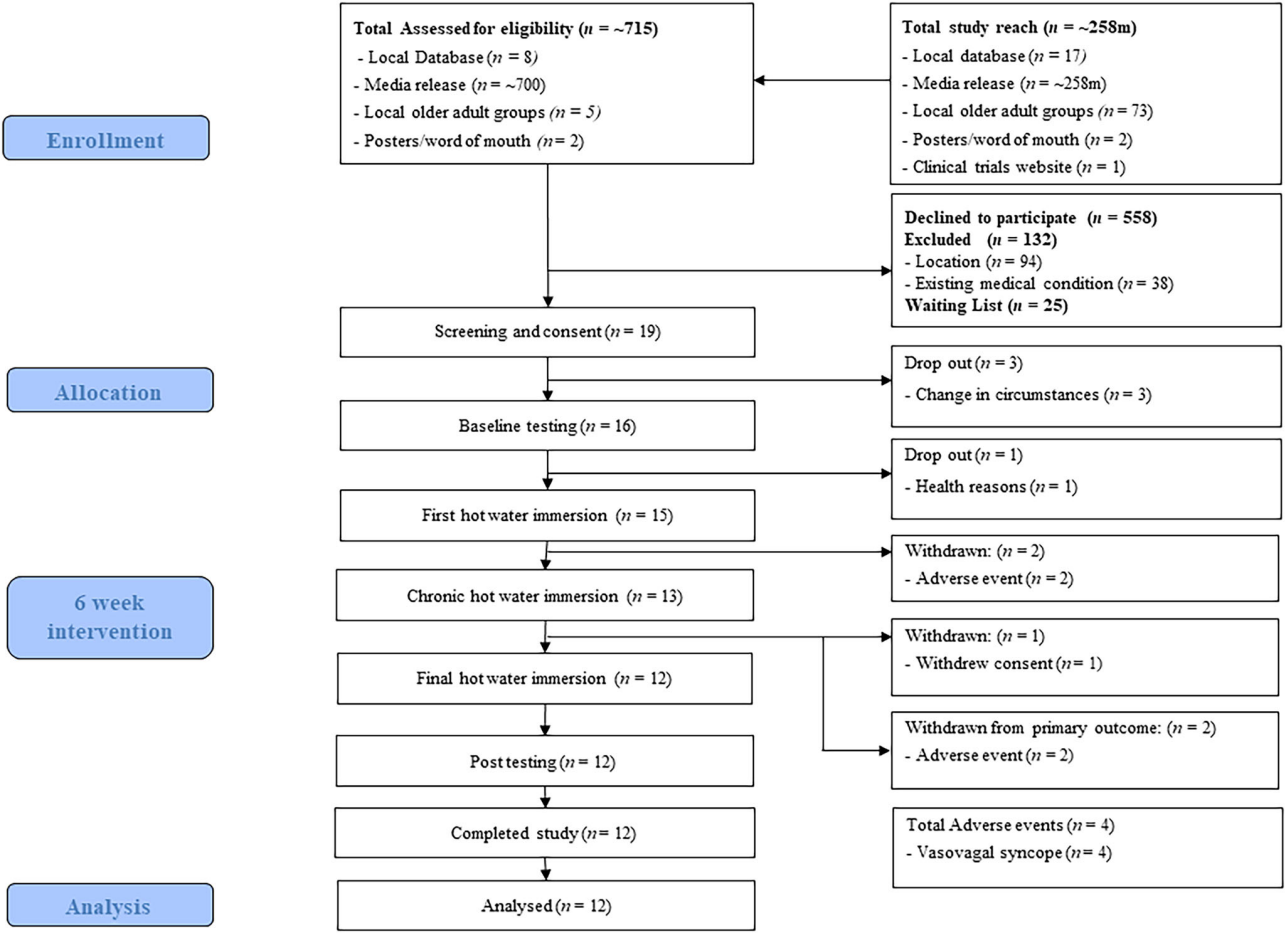
Participant flow through the trial.

Nineteen participants (Table 1) were recruited, of whom 12 completed the study (Figure 2). Attrition was due to change in personal circumstances (*n *= 3) and health reasons (*n *= 1) unrelated to the study, withdrawn consent (*n *= 1), and two adverse events (*n *= 2), in which both participants withdrew following vasovagal syncope during intravenous cannulation. Two additional participants were unable to complete the primary cognitive outcome measure due to adverse events (vasovagal syncope during HWI from orthostatic hypotension) but contributed to the other outcome measures. All participants who completed the study were Caucasian, removing any potential skewing of NIRS data due to skin pigmentation (Patel et al., 2024). The full anonymised dataset has been made freely available as supplementary material on our University repository (https://doi.org/10.17029/6ed6cf5d‐d598‐4a39‐8d07‐46a06441eb7f).

**TABLE 1:** Participant characteristics (*n* = 12).

Parameter	Value
Age (years)	69.2 ± 10.0
Males (*n*)	4
Females (*n*)	8
Height (cm)	169 ± 10
Mass (kg)	72.4 ± 16.8
BMI (kg m^−2^)	25.2 ± 4.1
SBP (mmHg)	131 ± 10
DBP (mmHg)	78 ± 7
Medications	
Alendronic acid (*n*)	1
Amitriptyline (*n*)	2
Beclometasone (*n*)	1
Finasteride (*n*)	1
Glucosamine (*n*)	1
Levothyroxine (*n*)	1
Simvastatin (*n*)	3
Solifenacin (*n*)	1
Tramadol (*n*)	1
Ventolin (*n*)	1
Zopiclone (*n*)	1

*Note*: Data are expressed as means ± SD unless otherwise stated. Abbreviations: BMI, body mass index; DBP, diastolic blood pressure; SBP, systolic blood pressure.

### 3.1 Rectal temperature

Participants (*n* = 12) completed 16 ± 2 HWI sessions during the study, achieving our target of 12–18 HWI sessions over the 6 weeks. One participant was unable to complete the final 5 min of one HWI session due to an adverse event (vasovagal syncope), and the full 60 min of immersion was completed for all other HWI sessions attended. For the 10 participants who completed cognitive performance assessments, *T*_rec_ at the start of the testing battery did not significantly differ between the first and final HWI sessions (first: 38.58 ± 0.20°C vs. final HWI: 38.66 ± 0.20°C; *t*_(9)_ = −1.041, *P* = 0.325, *d* = 0.41). Grand mean Δ*T*_rec_ per HWI session was 1.68 ± 0.14°C (*n* = 12). Resting *T*_rec_ significantly increased by 0.37°C at the end of the 6‐week intervention compared to baseline (first HWI: 36.66 ± 0.15 vs. final HWI: 37.03 ± 0.25°C, *t*_(11)_ = −4.712, *P* < 0.001, *d* = 1.79). Plasma volume change from baseline to 6 weeks was negligible (+0.2%). The grand mean time to reach *T*_rec_ of 38.5°C was 44.5 ± 6.09 min, and participants remained above this threshold for 29.0 ± 5.3 min, including time spent cooling following the 60 min HWI (across all sessions).

### 3.2 Cognitive performance following repeated HWI

**FIGURE 3:**
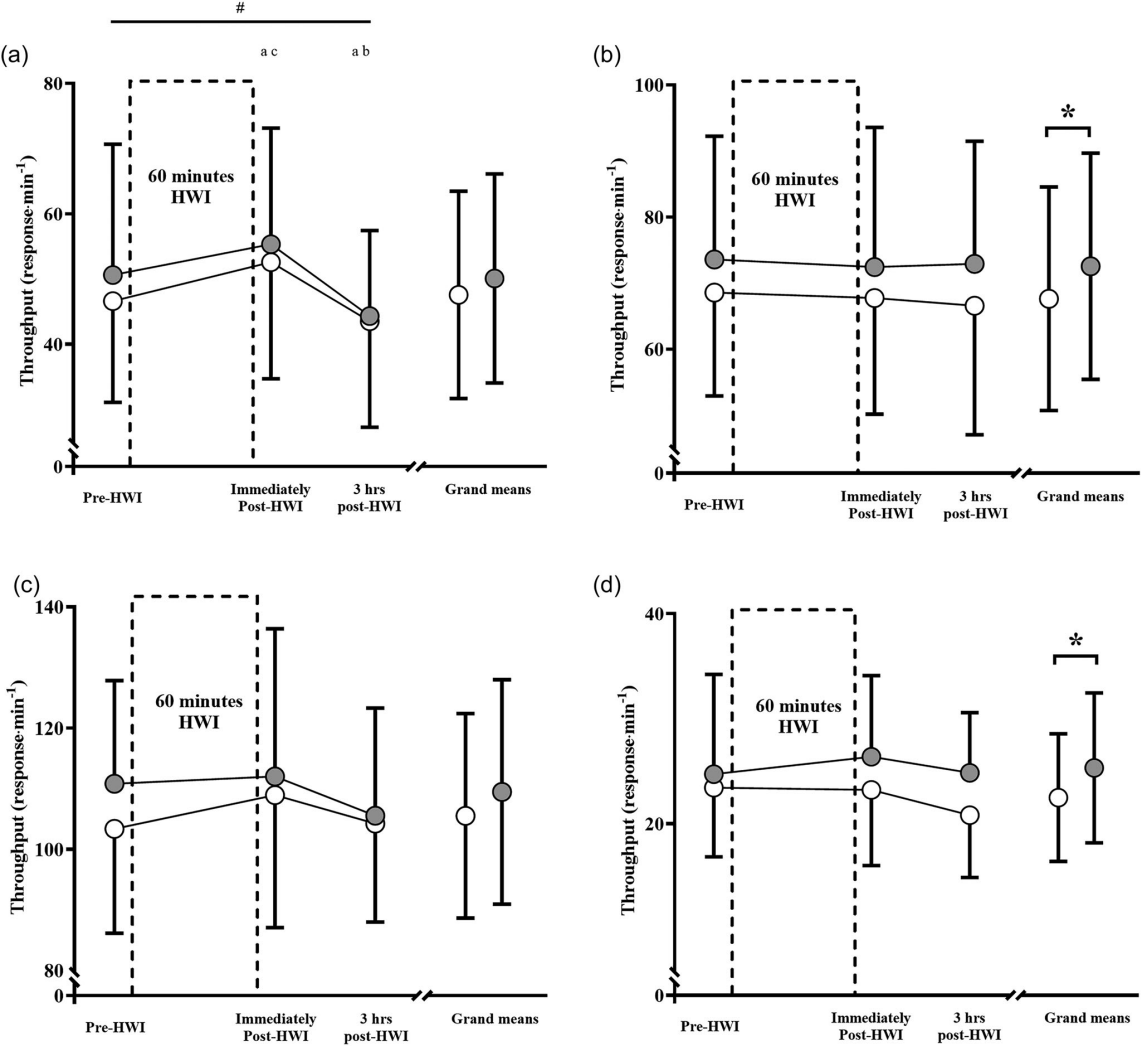
Acute and repeated cognitive performance. Means ± SD are presented for throughput and grand means for 2‐back (a), 1‐back (b), 2‐choice reaction time (c), and logical relations (d) completed at pre‐hot water immersion (HWI), immediately post‐HWI and 3 h post‐HWI (*n* = 10). Open circles represent the first HWI session and grey circles represent the final HWI session. *Significant difference between conditions (*P* < 0.05); ^#^significant main effect of time (*P* < 0.05); ^a^significant difference across time to pre‐HWI; ^b^Significant difference across time to immediately post‐HWI; ^c^significant difference across time to 3 h post‐HWI. The primary outcome, change in 2‐back performance between the first and final HWI session immediately post‐HWI, was assessed using a paired samples *t*‐test. Condition (first HWI and final HWI) × time (pre‐HWI, immediately post‐HWI and 3 h post‐HWI) was analysed using 2 × 3 linear mixed models and paired samples *t*‐test (grand means) for 2‐back, 1‐back, 2‐choice reaction time and logical reasoning.

There were no differences in throughput scores during the penultimate and final familiarisation sessions for any of the four cognitive tasks (2‐choice reaction: *t*_(9)_ = −1.215, *P* = 0.255, *d *= 0.30; logical relations: *t*_(9)_ = −2.137, *P* = 0.061, *d *= 0.64; 1‐back *Z* = −1.487, *P* = 0.160, *r *= −0.33; 2‐back: *t*_(9)_ = −1.050, *P* = 0.321, *d *= 0.20). Repeated HWI did not improve the primary outcome (2‐back throughput score immediately post‐HWI; first HWI: 53 ± 18 vs. final HWI: 55 ± 18 responses min^−1^; *t*_(9)_ = −1.049, *P* = 0.321, *d* = 0.15), and had no main effect on 2‐back (*F*_1,50_ = 2.578; *P* = 0.115, η_p_^2^ = 0.049) or 2‐choice reaction time (*F*_1,50_ = 3.294; *P* = 0.076, η^2^_p_ = 0.062) performance, but improved logical relations (*F*_1,50_ = 10.264; *P* = 0.002, η^2^_p_ = 0.170) and 1‐back (*F*_1,49.042_ = 5.486; *P* = 0.023, η^2^_p_ = 0.101) performance (Figure 3). Analysis of grand means showed an increase in throughput scores post repeated HWI for logical relations (first HWI: 22.48 ± 6.08 vs. final HWI: 25.31 ± 7.15 responses min^−1^; *t*_(9)_ = −2.813, *P* = 0.02, *d* = 0.43) and 1‐back (first HWI: 67.92 ± 16.93 vs. final HWI: 73.96 ± 17.15 responses min^−1^; *t*_(8)_ = −2.726, *P* = 0.026, *d* = 0.34) compared to pre HWI.

### 3.3 Cognitive performance following acute single exposure to HWI

An acute single exposure to HWI improved throughput scores in the 2‐back task (*F*_2,50_ = 13.593; *P* < 0.001, η^2^_p_ = 0.352) (Figure 3). *Post hoc* analysis revealed that 2‐back performance improved immediately post‐HWI compared to pre‐HWI (*P* = 0.008), while performance decreased at 3 h post‐HWI compared to pre (*P* = 0.018) and immediately post‐HWI (*P* < 0.001). No acute changes in throughput scores across time were observed for 2‐choice reaction time (*F*_2,50_ = 2.220; *P* = 0.119, η^2^_p_ = 0.082), logical reasoning (*F*_2,50_ = 1.686; *P* = 0.196, η^2^_p_ = 0.063) or 1‐back (*F*_2,49.042_ = 0.169; *P* = 0.845, η^2^_p_ = 0.007).

### 3.4 Cerebral oxygenation and CCA‐BF

**FIGURE 4:**
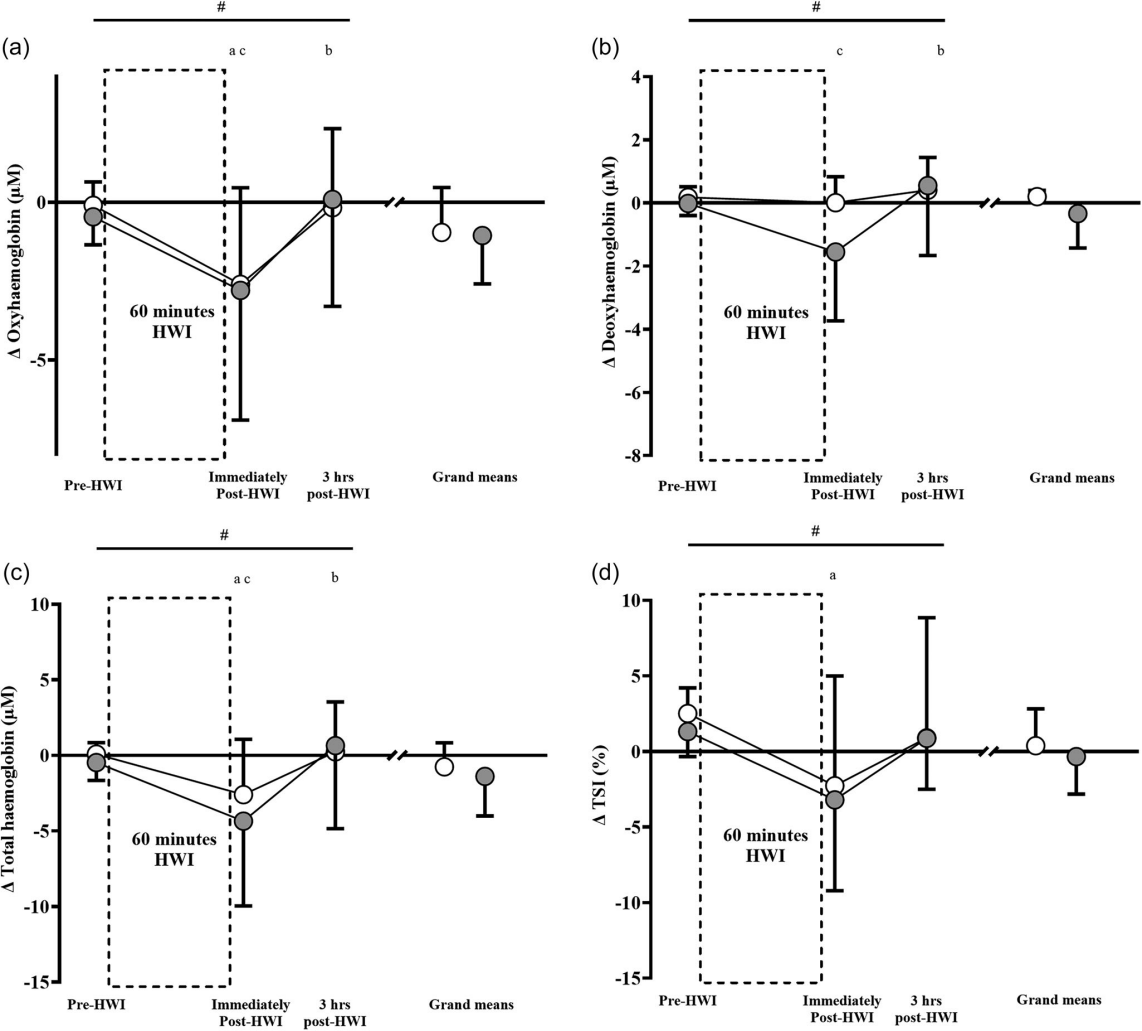
Cerebral oxygenation. Means ± SD are presented for cerebral oxygenation during cognitive tasks completed at pre‐hot water immersion (HWI), immediately post‐HWI and 3 h post‐HWI for (a) change in oxyhaemoglobin (ΔO_2_Hb), (b) change in deoxyhaemoglobin (ΔHHb), (c) change in total haemoglobin (ΔtHb), and (d) change in tissue saturation index (ΔTSI) (*n* = 8). Open circles represent the first HWI session and grey circles represent the final HWI session. ^#^Significant main effect of time (*P* < 0.05); ^a^significant difference across time to pre‐HWI; ^b^significant difference across time to immediately post‐HWI; ^c^significant difference across time to 3 h post‐HWI. Data were analysed using 2 × 3 linear mixed models [condition (first HWI and final HWI) × time (pre‐HWI, immediately post‐HWI and 3 h post‐HWI)] and paired samples *t*‐test (grand means) for 2‐back, 1‐back, 2‐choice reaction time and logical reasoning.

**FIGURE 5:**
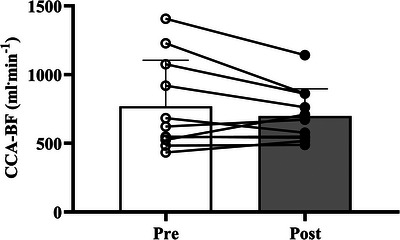
Common carotid artery blood flow. Bars and individual data points for common carotid artery blood flow (CCA‐BF) (*n* = 11). White bar with open circles represents pre‐HWI and grey bar with filled circles represents post‐HWI. Data were analysed using a paired samples *t*‐test.

An acute single exposure to HWI significantly reduced cerebral ΔO_2_Hb (*F*_2,38.173_ = 5.078; *P* = 0.011, η^2^_p_ = 0.210), ΔHHb (*F*_2,36.258_ = 4.308; *P* = 0.021, η^2^_p_ = 0.192), ΔtHb (*F*_2,37.391_ = 5.135; *P* = 0.011, η^2^_p_ = 0.215) and ΔTSI (*F*_2,37.293_ = 4.222; *P* = 0.022, η^2^_p_ = 0.185) across time (Figure 4). *Post hoc* analysis showed reductions immediately post‐HWI compared to baseline for ΔO_2_Hb (*P* = 0.011), ΔtHb (*P* = 0.013) and ΔTSI (*P* = 0.008). At 3 h post‐HWI, there were significant increases in ΔO_2_Hb (*P* = 0.007), ΔHHb (*P* = 0.007) and ΔtHb (*P* = 0.006) compared to immediately post‐HWI, with all NIRS values having returned to baseline (*P* > 0.05). Repeated HWI had no effect on grand means for ΔO_2_Hb (*Z* = −0.140, *P* = 0.945, *r* = −0.04), ΔHHb (*t*_(7)_ = 1.889, *P* = 0.101, *d* = –0.74), ΔtHb (*t*_(7)_ = 0.685, *P* = 0.515, *d* = −0.38) or ΔTSI (*t*_(7)_ = 0.562, *P* = 0.592, *d* = −0.28) during the acute single exposure in the final HWI session compared to the first HWI session. There were no significant correlations between ΔO_2_Hb, ΔHHb, ΔtHb or ΔTSI and any of the four cognitive tasks immediately post‐HWI in either the first or final HWI sessions (all *P* > 0.05). Repeated HWI did not significantly alter resting CCA‐BF (pre: 769 ± 334 vs. post: 697 ± 199 mL min^−1^; *t*_(10)_ = 1.443, *P* = 0.18, *d* = −0.26) (Figure 5).

### 3.5 Sleep

**FIGURE 6:**
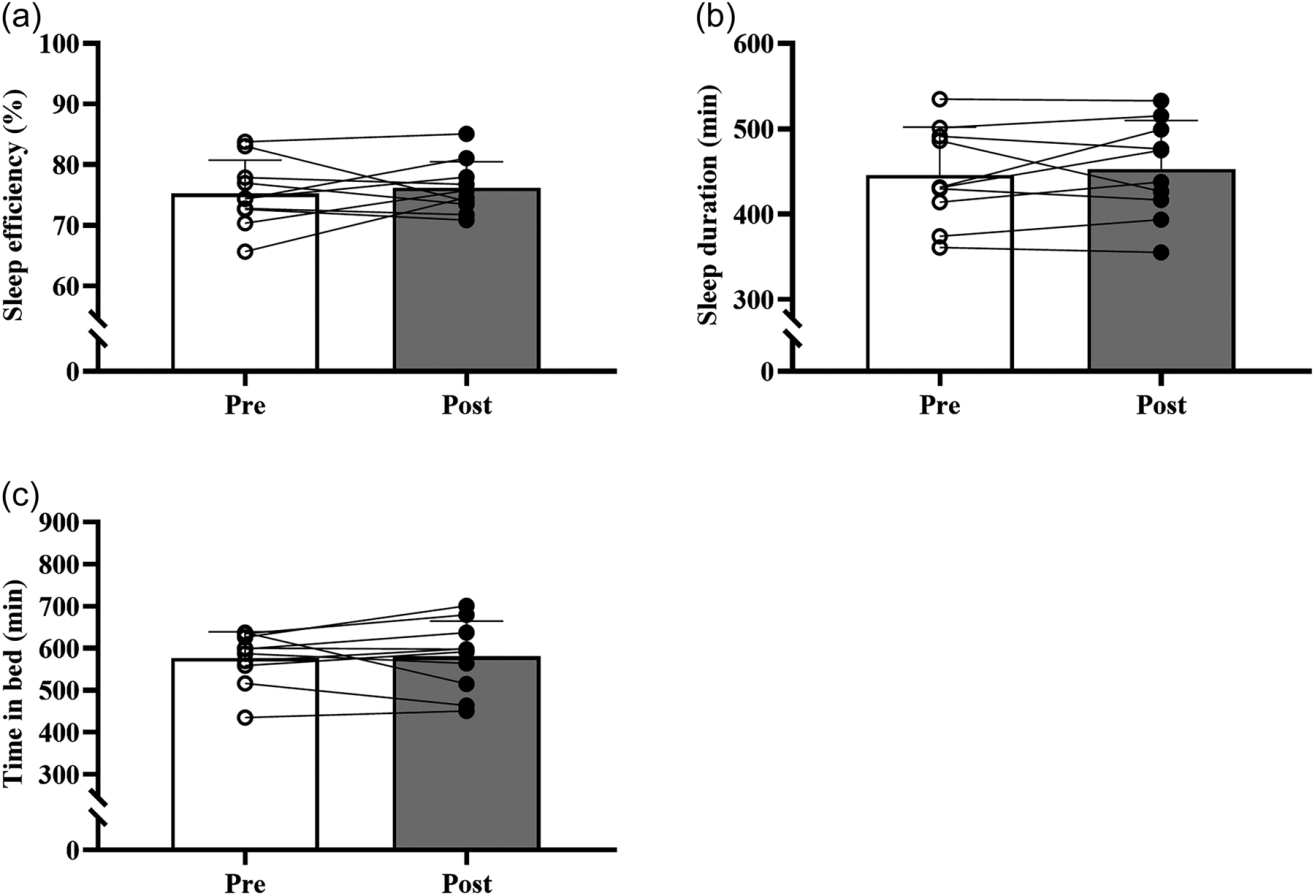
Sleep data. Bars and individual data points for sleep efficiency (a), sleep duration (b) and time in bed (c) for 7 days pre repeated hot water immersion (HWI) and during the final 7 days of the HWI intervention (*n* = 10). White bars with open circles represent pre‐HWI and grey bars with filled circles represent post‐HWI. Data were analysed using a paired samples *t*‐test.

There was no change in sleep efficiency (pre: 75.2 ± 5.5 vs. post: 76.2 ± 4.3 %; *t*_(9)_ = −0.548, *P* = 0.597, *d* = 0.19), sleep duration (pre: 445.4 ± 56.5 vs. post: 452.8 ± 56.6 min; *t*_(9)_ = −0.665, *P* = 0.523, *d* = 0.13) or time in bed (pre: 576.5 ± 62.1 vs. post: 580.2 ± 83.9 min; *t*_(9)_ = −0.201, *P* = 0.845, *d* = 0.05) between the 7‐day period before the 6 weeks’ repeated HWI and the final 7 days of the intervention period (Figure 6).

### 3.6 Biomarkers of neurodegeneration

**FIGURE 7:**
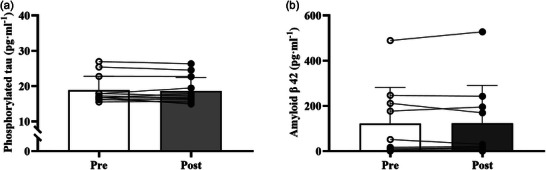
Biomarkers of neurodegeneration. Bars and individual data points for amyloid β42 (*n *= 10) (a) and phosphorylated‐tau (*n* = 12) (b) concentrations for pre and post‐ repeated hot water immersion (HWI). White bars with open circles represent pre‐HWI and grey bars with filled circles represent post‐HWI. Data were analysed using a Wilcoxon signed‐rank test.

Repeated HWI had no effect on plasma [Aβ42] (pre: 121.8 ± 160.4 vs. post: 123.0 ± 175.6 pg mL^−1^; *Z* = −0.357, *P* = 0.77, *r* = −0.08) (*n* = 10) or [p‐tau] (pre: 19.1 ± 4.2 vs. post: 18.7 ± 3.7 pg mL^−1^; *Z* = −0.706, *P* = 0.52, *r* = −0.14) (*n *= 12) (Figure 7). Two participants had unreadable plasma [Aβ42] values.

## 4 DISCUSSION

This is the first study to examine both the acute and repeated effects of HWI on cognitive performance, cerebral oxygenation and blood flow, sleep, and biomarkers of neurodegeneration in healthy older adults. The principal novel findings were that two to three HWIs per week for 6 weeks did not alter the primary outcome of 2‐back performance immediately post‐HWI in the final HWI session compared to the first HWI session. Repeated HWI improved logical reasoning and 1‐back performance following heating, but did not improve reaction time following heating, resting CCA‐BF, sleep or resting biomarkers of neurodegeneration. Acute HWI improved 2‐back performance and reduced cerebral oxygenation immediately post‐HWI compared to pre‐HWI, which then returned to baseline 3 h post‐HWI. Repeated HWI, however, did not negate these acute reductions in cerebral oxygenation immediately following the final HWI.

### 4.1 Cognitive performance

Six weeks of HWI improved logical reasoning ability and working memory (1‐back) performance in the ANAM task battery, contrasting with prior studies in young, healthy adults where repeated PHT did not significantly alter cognitive performance (Barry et al., 2022; Radakovic et al., 2007). The greater overall heating volume in the present study may have contributed to the observed benefits, but these effects may be limited to the specific cognitive domains tested in the present study. Additionally, previous repeated PHT studies were performed in young individuals who are unlikely to have cognitive impairment (Barry et al., 2022; Radakovic et al., 2007). Importantly, older adults typically exhibit age‑related cognitive decline (Harada et al., 2013) and our participants’ baseline scores in the cognitive tasks we tested (40th–75th percentiles) suggest capacity for improvement. A possible explanation for cognitive improvements could be improved thermal comfort, which can be increased following repeated heating (Sunderland et al., 2007), where decreased comfort reduces performance in cognitive tasks (Byrne et al., 2020). Whilst we did not examine thermal comfort levels during HWIs, our participants did not display physiological signs of heat acclimation and it is possible that cognitive improvements may have been independent of comfort levels.

We showed that working memory performance (2‐back) improved immediately post‐HWI both in the first and final HWI. This is in contrast to previous acute PHT work by Schlader et al. (2015), who observed no changes in memory performance, but saw faster reaction time in healthy older adults when deep body temperatures were raised by 1.0°C. We also demonstrate that 1‐back performance, 2‐choice reaction time and logical reasoning did not change across time following acute HWI in either the first or the final HWI. Our findings in the final HWI are similar to Radakovic et al. (2007) who found that, compared to a control group, repeated PHT prevented decrements in cognitive performance after an exertional heat stress test.

Schmit et al. (2017) proposed an inverted‑U relationship between deep body temperature and cognitive performance, with improvements up to deep body temperatures of ∼38.5°C, followed by a plateau then a decline at ∼39°C. Despite our participants’ deep body temperatures being ∼38.6°C at the time of taking the cognitive tests, only the 2‐back task improved following acute heat stress. It should be noted, however, that aside from the study by Schlader et al. (2015), the data reviewed by Schmit et al. (2017) were derived from young, healthy populations. Our results in conjunction with those of Schlader et al. (2015) suggest that when exposed to heat stress, cognitive responses in older adults act differently to younger populations. Future studies investigating the interactions between thermal load and cognitive tasks of varying complexity are warranted in older adults to elucidate this relationship in ageing populations.

### 4.2 Cerebral oxygenation

Acute HWI reduced ΔtHb and ΔTSI immediately post‐HWI compared to pre‐HWI, with decreased ΔtHb primarily driven by a concurrent decrease in ΔO_2_Hb. The findings are consistent with the Bohr effect, indicating a greater local O_2_ utilisation; however, metabolic or positron emission tomography (PET) data would be needed to confirm this. These small reductions seen in cerebral oxygenation and likely increased O_2_ utilisation appear to subsequently return to baseline at 3 h post‐HWI. We also found that repeated HWI did not attenuate these decreases in oxygenation in response to acute heat stress. Only one study has investigated cerebral NIRS responses to acute passive heating, demonstrating similar reductions in tHb, but no changes in TSI in young, healthy individuals (Morrison et al., 2009), though this study had a small sample size (*n* = 5) and may have lacked adequate statistical power. Despite significant reductions in oxygenation and subsequent return to basal levels in the present study, the magnitude of reductions may not have been enough to directly influence cognitive performance. Further interrogation of our data showed that cognitive performance immediately post‐HWI was not correlated with cerebral oxygenation (i.e., TSI) in our study population (all *P* > 0.05), including the improvements in the 2‐back task which occurred when cerebral oxygenation was decreased (i.e., immediately following heating).

Given that reductions in cerebral oxygenation from different stressors, including exercise (Mekari et al., 2015) and hypoxia (Friend et al., 2019; Williams et al., 2019), have been shown to be associated with decreases in cognitive performance in young adults, it is surprising that we did not see similar decreases following HWI; rather, cognitive performance was either unchanged or improved. Therefore, there is likely a differing relationship between cerebral oxygenation and cognitive performance related to both a HWI stimulus and age, potentially linked to increases in oxygen extraction, though this appears to be preserved at rest (Lin et al., 2022) and during exercise (Braz & Fisher, 2016). Together, this suggests that the mechanism(s) by which cognitive performance improved may not be solely due to alterations in cerebral oxygenation per se in this cohort and after this intervention.

Although we did not measure brain activity, a possible explanation for improvements in cognitive performance despite reductions in cerebral oxygenation could relate to the manner of older adults’ brain activation in tests of cognitive performance. Young adults performing working memory tasks primarily activate a single prefrontal region of the brain (Reuter‐Lorenz et al., 2000). Older adults, however, appear to increase brain activation by recruiting resources from both sides of the brain as a compensatory mechanism for decreases in hippocampal volume to maintain cognitive performance (Park & Reuter‐Lorenz, 2009). Additionally, it has been shown that older individuals with the poorest memories tend to display the greatest levels of brain activation to complete the tasks (Persson et al., 2006). Therefore, as our participants did not have any clinically diagnosed cognitive impairments, it is possible the combined load of the cognitive tasks and heat stress induced overcompensated brain activation in order to perform the cognitive tasks. Future work investigating the unexpected observed divergent responses to HWI using functional MRI or PET in conjunction with heating and tests of cognition would be needed to confirm this.

### 4.3 CCA‐BF

Our data indicate that repeated HWI three times per week for 6 weeks did not change resting CCA‐BF in older adults. Previous HWI studies showed acute (Gibbons et al., 2021; Worley et al., 2021) and repeated HWI (Bailey et al., 2016) increases in CBF in younger individuals, potentially via concurrent changes in endothelial function (Bailey et al., 2016). Therefore, 6 weeks may not have been sufficient to improve endothelial function of the carotid artery in older individuals. Thus, chronic improvements in cognitive performance in our study appear to be independent of any effects on resting CCA‐BF in healthy older adults. Indeed, there is evidence to suggest that cognitive performance could be more affected by changes in brain volume (Poels et al., 2008) or cerebral metabolism (Ogoh, 2017) rather than strictly CBF. Future work should include these as a potential mechanism for improvements in cognitive function following repeated HWI, though based on previous long term exercise‐based work (Erickson et al., 2011), chronic changes likely would require longer intervention periods than the 6 weeks in the present study.

### 4.4 Sleep

We showed that repeated HWI did not affect sleep efficiency, sleep duration or time in bed in our cohort of healthy older adults. While previous HWI studies exploring any impact upon sleep are limited, studies that have shown improvements have implemented HWI in close proximity to sleeping (Bunnell et al., 1988; Liao, 2002). It is possible that to improve sleep quality, HWI sessions needed to be in the evening; however, this requires further investigation and should be approached with caution, as raised body temperature resulting from evening bathing could disrupt the circadian rhythm body temperature regulations and impair sleep quality (Osborne et al., 2023). All HWI visits in the present study took place during daytime hours (09.00–17.00 h), and we show that repeated HWI in the morning or daytime hours did not cause any detriments to sleep in our cohort. Chronic studies have also shown improved sleep quality in older adults following exercise (Durcan et al., 2014) or resistance training (Gambassi et al., 2015) interventions; however, the length of these interventions were 12–16 weeks, considerably longer than the 6 weeks in the present study. Therefore, it is possible that the duration of the present study was not long enough to elicit any changes in sleep parameters. Longer interventions that also focus on the timing of bathing are therefore warranted to determine if repeated HWI can improve sleep in older adults.

### 4.5 Biomarkers of neurodegeneration

For the first time, we examined the effect of repeated HWI on plasma biomarkers of neurodegeneration. We showed that 6 weeks’ HWI had no effect on [Aβ42] or [p‐tau]; however, the large variability in our data for [Aβ42] suggests heterogeneity in our healthy cohort without known cognitive impairment with low likelihood in detecting meaningful differences. Our results, therefore, may not be generalisable to a wider population of older adults, and future studies investigating neurodegenerative biomarkers may benefit from pre‐screening for baseline levels to ensure a homogeneous study population. Additionally, the 6‐week duration of our study likely was not adequate to improve [Aβ42] and [p‐tau]. A recent review of the exercise literature demonstrated that various exercise interventions spanning 8 weeks up to 1 year did not reduce [Aβ] (Pucci et al., 2024) or p‐tau (Steen Jensen et al., 2016). Thus, it is possible that neither exercise nor HWI may be suitable interventions to reduce concentrations of these biomarkers of neurodegeneration.

### 4.6 Limitations

The study employed a pre–post design, whereas a randomised crossover would be the gold standard to ensure that improvements in cognitive performance were not due to the occurrence of a learning effect. Despite this, our familiarisation protocol was in line with manufacturer guidelines and validation data showing good or excellent intraclass correlation coefficients (0.65–0.95) (ANAM, 2015; Vincent et al., 2018). There were no statistical differences between throughput scores for the final two ANAM familiarisation trials undertaken for any of the four cognitive tasks, indicating that participants were sufficiently familiarised with the cognitive tasks before undertaking the experimental trials. The interpretation of our results for cerebral oxygenation during the cognitive tasks should be approached with some caution, as to our knowledge the Portalite has not been validated against the gold standard arterial‐internal jugular venous $P_{O_{2}}$ sampling following heat stress in older individuals, and may not accurately reflect changes in blood flow (Hoiland et al., 2020). However, due to the nature of the experimental conditions, a non‐invasive technique was necessary to conduct these measurements. Additionally, we did not record skin blood flow in the forehead, which could have impacted the findings. While care was taken in the analysis of NIRS data to limit the influence of extracranial signal contamination, we were unable to fully remove the data recorded at the 30 mm depth, as the T3 data included readings from all three depths (i.e., T1, T2 and T3).

A further limitation was that due to participant availability, timing of testing in the final HWI session was not identical to the first HWI. This could have led to increased resting *T*_rec_ (i.e., lack of heat acclimation) following repeated HWI due to the diurnal variations that occur in body temperature across the day. We also did not perform a power calculation for any secondary outcome measures, and therefore findings for these variables may be underpowered and not representative of a true effect taking place. We also did not collect any demographic data (e.g., education levels) from our participants, which is highly predictive of executive function (Caballero et al., 2020), and therefore our data may not be representative of the wider older adult population. Whilst participants stated that they had no diagnosed neurological conditions, individuals were not prescreened for mild cognitive impairment prior to participation, and therefore we cannot preclude that some of our participants may have been cognitively impaired. Our results are additionally only applicable to a laboratory study, and do not account for any safety risks of adverse events (i.e., vasovagal syncope) from HWI in a more ecologically valid setting.

In the current study we conducted only ultrasound measurements of the CCA, and therefore we are unable to determine blood flow distribution specifically through the internal carotid, external carotid and vertebral arteries, of which the external carotid artery supplies blood to non‐brain regions (e.g., face, neck). Previous HWI studies showing changes in CBF (Bailey et al., 2016; Gibbons et al., 2021; Worley et al., 2021) measured middle cerebral artery velocity using transcranial Doppler, which would have provided a valid measurement of CBF. Additionally, we did not measure CCA‐BF during HWI or immediately following the first and last HWI sessions, which may have provided further insight into the relationship between blood flow, oxygenation and cognitive performance. A further limitation was that we did not measure Aβ42 and p‐tau directly from PET imaging or cerebrospinal fluid collection. Despite this, plasma measurements of [Aβ42] and [total tau] have previously been correlated with PET and cerebrospinal fluid levels (Nakamura et al., 2018) and dementia risk (Pase et al., 2019), respectively, making it a suitable, minimally invasive and cost‐effective alternative for providing an indication of brain levels of these parameters.

### 4.7 Conclusion

The present study is the first to assess the effects of repeated HWI on cognitive performance, cerebral oxygenation, CCA‐BF, sleep and biomarkers of neurodegeneration in healthy older adults. Overall, repeated HWI improved cognitive domains of logical reasoning and working memory performance; however, this appears to be unrelated to concurrent reductions in cerebral oxygenation. Additionally, acute HWI improved working memory performance (2‐back) independent of repeated HWI, but did not improve reaction time or logical reasoning. Therefore, repeated HWI could be used as a potential method to improve select aspects of cognitive function in older adults. Future trials with a control group, longer HWI interventions and direct examinations of brain activity and CBF (e.g., fMRI, PET, transcranial Doppler) are warranted to elucidate the mechanisms behind improvements in cognitive performance in healthy, older adults.

## AUTHOR CONTRIBUTIONS

Daniel D. Piccolo, Anthony I. Shepherd, Jo Corbett and Zoe L. Saynor conceived and designed the research. Daniel D. Piccolo, Anthony I. Shepherd, Mohammad G. A. Alnajjar, Luke Hudson Poppy A. Marsh and Veronika Praskacova collected the data. Daniel D. Piccolo, Anthony I. Shepherd, Thomas B. Williams, Harry S. Mayes, Melitta A. McNarry, and Kelly A. Mackintosh analysed the data. Daniel D. Piccolo, Anthony I. Shepherd, Jo Corbett and Zoe L. Saynor interpreted results of experiments and trial. Daniel D. Piccolo and Anthony I. Shepherd prepared figures. Daniel D. Piccolo, Anthony I. Shepherd, Jo Corbett and Zoe L. Saynor drafted the manuscript. Daniel D. Piccolo, Anthony I. Shepherd, Jo Corbett, Zoe L. Saynor, Mohammad G. A. Alnajjar, Luke Hudson Poppy A. Marsh, Veronika Praskacova, Joseph T. Costello, Thomas B. Williams, Thomas J. James, Harry S. Mayes, Michael Tipton, Maria Perissiou, Melitta A. McNarry, and Kelly A. Mackintosh edited and revised the manuscript. All authors approved the final version of manuscript and agree to be accountable for all aspects of the work in ensuring that questions related to the accuracy or integrity of any part of the work are appropriately investigated and resolved. All persons designated as authors qualify for authorship, and all those who qualify for authorship are listed.

## CONFLICT OF INTEREST

None declared.

## Data Availability

The full anonymised dataset has been made freely available as supplementary material on our University repository (https://doi.org/10.17029/6ed6cf5d‐d598‐4a39‐8d07‐46a06441eb7f). For the purpose of open access, the authors have applied a Creative Commons Attribution (CC‐BY) licence to any author accepted manuscript version arising from this submission.
